# Supplementary material for: PiCCL: A lightweight multiview contrastive learning framework for image classification
Source: PLoS One. 2025 Aug 25;20(8):e0329273. doi: 10.1371/journal.pone.0329273 (PMC12377561; doi:10.1371/journal.pone.0329273)
Supplement: Attachment — Submitted filename: Reviewer Attachment.docx [file pone.0329273.s001.docx]

**Manuscript Number:** PONE-D-24-42971R1

**Manuscript Title:** PiCCL: a lightweight multiview contrastive learning framework for image classification

The article was reviewed under the title "PiCCL: a lightweight multiview contrastive learning framework for image classification". Overall, the focus of this study is good. However, I would like to offer several recommendations that the authors might consider beneficial in the process of revising their manuscript:

1) I suggest adding a clear research objective and research questions in the introduction section and specify what the main research problem or hypothesis is addressed.

2) Please add more recent references in the related works section. Certainly, there has been more recent (within the last two years) research on this topic published in information science and/or computer science outlets. An academic search on the topic (using keywords from the manuscript's title) shows that there is recent work in this area. Therefore, authors must update their literature review.

3) Starting from the previous works, I suggest introducing a table to summarize the most recent works and to highlight the novelty of the proposed work.

4) The manuscript only provides the structure of network, it is necessary to explain why these networks can work effectively and to show the concrete parameters.

5) Although PiCCL is a multiview contrastive learning algorithm, it is required to provide technical details of the proposed methods as much as possible and in-depth explanations of method selection process.

6) I suggest adding a table that contains various augmentation techniques with parameters and values.

7) The authors should need to provide details about dataset.

8) How well does the model perform on purely real-world datasets without augmentation?

9) The captions for figures and tables could be expanded to guide the reader better. For example, could the authors explain the significance of each figure in the context of their results?

10) The structure of Primary Component Contrastive learning network is innovative. However, it is better to analysis the computational complexity of this network in the revised manuscript.

11) It is suggested to conduct statistical tests for the proposed system. For example, see Section 4.5 in paper [a] and the author could cite this paper for reference.

[a] Biswas, S., Mostafiz, R., Uddin, M.S. and Paul, B.K., 2024. XAI-FusionNet: Diabetic foot ulcer detection based on multi-scale feature fusion with explainable artificial intelligence. *Heliyon*, *10*(10), p.e31228.

12) I think, the authors should introduce a comparison table to compare the proposed work with other existing works that contains dataset size, methods, performance etc.

13) The authors could cite following papers for data augmentation if found relevant:

i. S. Biswas, R. Mostafiz, B. K. Paul, K. M. Mohi Uddin, M. M. Rahman, and F. N. U. Shariful, “DFU_MultiNet: A deep neural network approach for detecting diabetic foot ulcers through multi-scale feature fusion using the DFU dataset,” *Intelligence-Based Medicine*, vol. 8, p. 100128, 2023.

ii. S. Biswas, R. Mostafiz, B. K. Paul, K. M. M. Uddin, Md. A. Hadi, and F. Khanom, “DFU_XAI: A Deep Learning-Based Approach to Diabetic Foot Ulcer Detection Using Feature Explainability,” *Biomedical Materials & Devices*, pp. 1-21, Mar. 2024.

iii. F. Khanom, S. Biswas, M. S. Uddin, and R. Mostafiz, “XEMLPD: An Explainable Ensemble Machine Learning Approach for Parkinson Disease Diagnosis with Optimized Features,” *International Journal of Speech Technology*, pp. 1-29, 2024.
