# Supplementary material for: PiCCL: A lightweight multiview contrastive learning framework for image classification
Source: PLoS One. 2025 Aug 25;20(8):e0329273. doi: 10.1371/journal.pone.0329273 (PMC12377561; doi:10.1371/journal.pone.0329273)
Supplement: Attachment — Submitted filename: response_to_reviewers.docx [file pone.0329273.s002.docx]

Dear Editor, thank you again for reviewing our work. We have addressed each one of your comments as best as we can. Below are your comments followed by our responses.

- The authors should cite all related works in the Table 1.

Response: We have added citation (links) to the table.

- In this research, the authors chose multiview contrastive learning algorithms, they should explain in detail why they did that. Also they used Siamese networks, they should give a reason to do that.

Response: During the last revision, we added a few sentences explaining our objectives, for example, this sentence from introduction paragraph 3: “The search for better SSL algorithm is an ongoing task. Our research objective has been to develop methods that performs well, don’t require large batch size, and can run on limited hardware.” We chose to innovate on CL because we think it is very popular, has great potential, and aligns with our objective. And MCL felt like a logical extension toward realizing our objective. Onto the question of why we choose Siamese network, The definition of Siamese network is not strictly defined. We like to think that networks containing multiple branches of both the same structure and weights are Siamese networks, while networks containing branches of different weight or structure should be categorized as pseudo-Siamese networks. The branching network structure is likely an integral part of CL, as contrastive learning learns from the contrast of 2 representations. For our work we avoided pseudo-Siamese network because we want to keep our model as simple as possible in hopes of improving generalizability. Also, pseudo-Siamese networks might require more computation or use more memory, one example is SimSiam in table 6.

- They said that “image augmentation methods is crucial for the quality of representation learning . A good augmentation should retain as much relevant information as possible while altering the rest”, so they should explain in detail why they chose the image augmentation process used by Barlow-Twins.

Response: We used it because (1), our method is inspired by barlow-twins, and (2), it provided better results than the image augmentation process of SimCLR. The choice of augmentation method is crucial for getting the absolute best accuracy, but it doesn’t effect the relative performance between methods by much, so it shouldn’t effect the validity of our comparisons.

- The main contribution is a loss function designed for PiCCL, they should explain it in detail.

Response: We have expanded Section 3.2, which provides explanations for the loss function.

- They chose L2 normalized embedding vector in their loss function, why did they do that.

Response: Dot product of the L2 normalized vectors (cosine similarity) is a widely used method in CL, including SimCLR and Barlow-Twins. We initially avoided the term “cosine similarity” because it provides an extra burden to the reader, but now we think it explains why L2 normalization is required as well as provides more intuition, therefore we have added it in.

- What reason did their proposed model PiCCL (8) get the best result, could they explain in detail.
Response: We don’t have definitive proof on why PiCCL(8) get the best results, but we do have some logical guesses. Introduction paragraph 4 provides some intuition for PiCCL, to put it briefly, more views (branches) can reduce the stochastic discrepancy, similar to the central value theorem. Section 5.1 discusses the performance of our algorithm, which we inferred the positive relationship between the number of views and the accuracy.

- Take a look the result in Table 6, PiCCL (8) is not good in both Time per epoch and Memory, what is a reason?

Response: This is because more views mean more data to remember and process.

- In comparison, they only compared with SimCLR ([12]:2021), Barlow-Twins ([14]:2021) and SimSiam ([13]:2020)), how did they get the result of other works?
- They should do more experiments, analyze the result in detail and compare with advanced methods in recent year.

Response: Sorry for altering the order of your comments, but we think these 2 questions should be answered together. We have added cifar10 and cifar100 results for E-SSL (section 4.2). We have put in a lot of effort trying to make more comparisons with other methods, but we were unable due to a combination of hardware constraint, code availability, and representativeness considerations.

One of PiCCL’s innovations is the new loss function, or regularization constraint. The network structure is simple (encoder + projector, identical branches), and the specific choice of encoder network is less important. We prioritize methods that also have these features when picking candidates for comparison. These include methods listed in table 1, the three most important being SimCLR, Barlow-Twins, and CMC. Although there are many SSL image classification frameworks in recent years, most innovate on network structure, or specific use cases. A survey [1] published in 2024 could somewhat support our claim, in the table summarizing CL methods, we see that many of them shares the same few loss functions.

Our team has a single workbench containing a single RTX-3090 (24GB Vram), which only allow us to test small models at small batch sizes. Since we can’t test PiCCL on the same tests conducted in SimCLR and Barlow-Twins, we must retest the competing methods on our hardware with our set of parameters, which is time consuming and challenging. And more importantly, we cannot test methods where the source code isn’t published, this is the reason why we didn’t test K-shot or LOOC.

Upon receiving your comment, we were determined to add both CMC and E-SSL to the comparison. However, these 2 methods are structurally very different from our program and cannot be adapted easily. CMC differ from PiCCL in that it uses non identical network branches (both structure and weights), and also uses a memory bank to store representations of every samples. E-SSL differs from PiCCL in that its input views have 2 sizes, the larger one is for contrastive learning, the smaller one is for rotation prediction. Due to these differences, we attempt to update their code to run our test rather than changing our code to accommodate these methods. CMC’s published code is written for ImageNet100 and ImageNet, to test it on cifar10, cifar100, and stl10, we altered the encoder the same way we conduct other tests. CMC on default uses a special image augmentation module that converts RGB image to Lab format so we couldn’t use the Barlow-Twins transformation from other tests, only the size parameter was altered. No other parameters was changed to preserve originality. The training results were suboptimal for all datasets, averaging more than 10 percentage points lower than other methods. A similar problem was encountered for E-SSL. E-SSL was written for cifar10 and ImageNet, so results for cifar10 were good, adapting it to cifar100 also yield good results. However, for stl10 the same performance degradation was encountered. We suspect these suboptimal performance metrics are the results of unoptimized parameters and require careful parameter tuning to fix. Regardless of the reason, we do not think these results are a fair representation of the creator’s work.

For this revision, because the other test results are significantly lower than we anticipated, we only added E-SSL’s performance on cifar10 and cifar100. Although we included them, we do not think they can be held up to the same standards as the other test results, because it uses a drastically different network, as well as a different image augmentation module. If you also think it’s better not to include these results, we can remove them in the next revision.

[1] Hu, H., Wang, X., Zhang, Y., Chen, Q., & Guan, Q. (2024). A comprehensive survey on contrastive learning. *Neurocomputing*, *610*, 128645. https://doi.org/10.1016/j.neucom.2024.128645
